# Dynamics of angiogenesis in ischemic areas of the infarcted heart

**DOI:** 10.1038/s41598-017-07524-x

**Published:** 2017-08-02

**Authors:** Koichi Kobayashi, Kengo Maeda, Mikito Takefuji, Ryosuke Kikuchi, Yoshihiro Morishita, Masanori Hirashima, Toyoaki Murohara

**Affiliations:** 1 Department of Cardiology, Nagoya University Graduate School of Medicine, Nagoya, 466-8560 Japan; 2 Department of Medical Technique, Nagoya University Hospital, Nagoya, 466-8560 Japan; 3 Division of Vascular Biology, Kobe University Graduate School of Medicine, Kobe, 650-0017 Japan

## Abstract

Cardiomyocytes are susceptible to apoptosis caused by hypoxia during the acute and subacute phases of myocardial infarction (MI). Angiogenesis can reduce MI-induced damage by mitigating hypoxia. It has been speculated that the ischemic border zone is a unique area rescued by angiogenic therapy. However, the mechanism and timing for new vessel formation in the mammalian heart following hypoxia are unclear. Identifying targets that benefit from angiogenesis treatment is indispensable for the development of revolutionary therapies. Here, we describe a novel circulatory system wherein new vessels develop from the endocardium of the left ventricle to perfuse the hypoxic area and salvage damaged cardiomyocytes at 3–14 days after MI by activating vascular endothelial growth factor signaling. Moreover, enhanced angiogenesis increased cardiomyocyte survival along the endocardium in the ischemic zone and suppressed ventricular remodeling in infarcted hearts. In contrast, cardiomyocytes in the border zone’s hypoxic area underwent apoptosis within 12 h of MI, and the border area that was amenable to treatment disappeared. These data indicate that the non-perfused area along the endocardium is a site of active angiogenesis and a promising target for MI treatment.

## Introduction

Myocardial infarction (MI) is a leading cause of death in westernized countries. Early thrombolysis and subsequent catheter intervention therapy during the acute phase reduce the size of the infarct and improve the prognoses of MI patients^1, 2^. However, a subset of patients requires ventricular assistance devices or heart transplantations, because of the extreme impairment of cardiac function. New treatments that use angiogenic or anti-apoptotic factors, and cell-based therapies are expected to alleviate heart damage. However, clinical trials involving vascular endothelial growth factor (VEGF) or fibroblast growth factor (FGF) have not yielded the expected results^3–5^; thus, no effective or innovative therapies have been developed for MI since catheter intervention became a widely used treatment. One reason for the lack of adequate alternative therapies is that the target area and cells to be salvaged in the infarcted heart have not been identified, partly because MI patients typically have a variety of disease conditions. Most newly developed therapies target the interface between the perfused and non-perfused areas of the heart, and they attempt to inhibit cardiomyocyte apoptosis or induce angiogenesis. However, the physiological events that occur in the MI border zone are not well understood. The spatial and temporal identification of the cells that are amenable to treatment is essential for the development of future and more effective therapies for MI.

## Results

### New vessels develop from the endocardium

We observed that a few layers of cardiomyocytes along the endocardium of the infarct area survived the chronic phase of MI in a mouse model. The mechanism by which the cells in this area can survive without blood perfusion from the coronary artery was investigated by analyzing lectin and pimonidazole hydrochloride (hypoxyprobe) staining. A hypoxyprobe forms adducts with thiol groups on proteins, peptides, and amino acids in live and hypoxic cells at <10 mmHg O_2_, but it is not incorporated into dead cells^6^. The mice were perfused with lectin to label the microvessels before they were sacrificed, and hypoxyprobe staining was used to detect the cells in the hypoxic areas in which the partial pressure of O_2_ was <10 mmHg as opposed to 15–20 mmHg in normal heart tissue.

**Figure 1:**
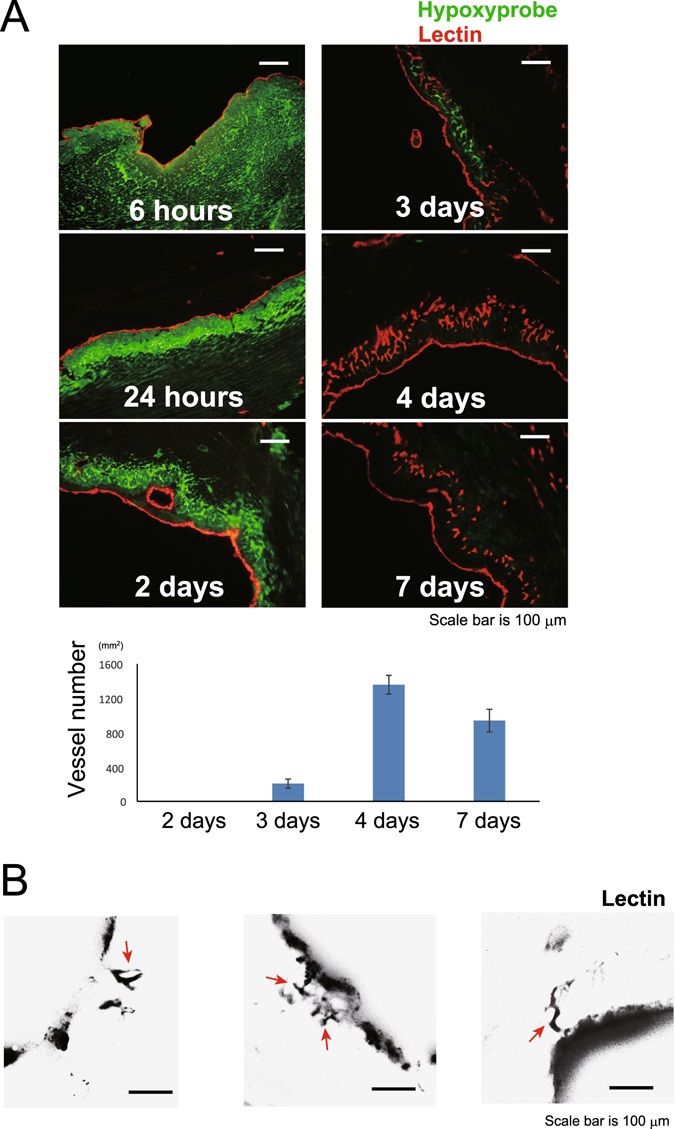
Angiogenesis develops through the endocardium from the left ventricle in ischemic areas of the infarcted heart. (**A**) Non-perfused areas within 100–150 μm of the endocardium were positively for the hypoxyprobe between 6 hours and 2 days after myocardial infarction induction. On day 3, perfused vessels emerged in this area, accompanied by a concomitant decrease in the hypoxyprobe staining. New vessels developed along the endocardium, and no hypoxic areas were detected after day 4. Scale bar, 100 μm. (*n* = 3 areas per time point) (**B**) Primitive vessels (arrows) which developed from the endocardium were observed on day 3. Vessels were stained with perfused lectin. Scale bar, 100 μm.

We detected cells that had survived in the area that was not perfused with blood along the endocardium after MI induction. Non-perfused areas which are negative for lectin staining had CD31 potisive endothelial cells of non-perfused vessels after 6 hours after MI induction (Fig. S1A). Non-perfused areas within 100–150 μm of the endocardium were positively labeled with the hypoxyprobe from 6 h to 2 days after MI induction. Non-perfused area, which are negative for hypoxyprobe, was stained with caspase and TUNEL positively (Fig. S1B, S1C). These data mean that the cardiomyocytes in this area die within 3 to 6 hours after MI induction. Part of the area, which was within 100–150 μm of the endocardium, lost the hypoxyprobe signal on day 3, at which time new vessels had started developing from the endocardium. The hypoxyprobe staining weakened and then disappeared in the area that was perfused with new vessels from the left ventricle (LV) (Fig. 1A), which increased dramatically over a broad area between days 3 and 4, and primitive bud-like vessels sprouted from the endocardium within the ischemic area of day 3 tissue (Fig. 1B).

### A plexus of primitive vessels from the endocardium alters the morphology

**Figure 2:**
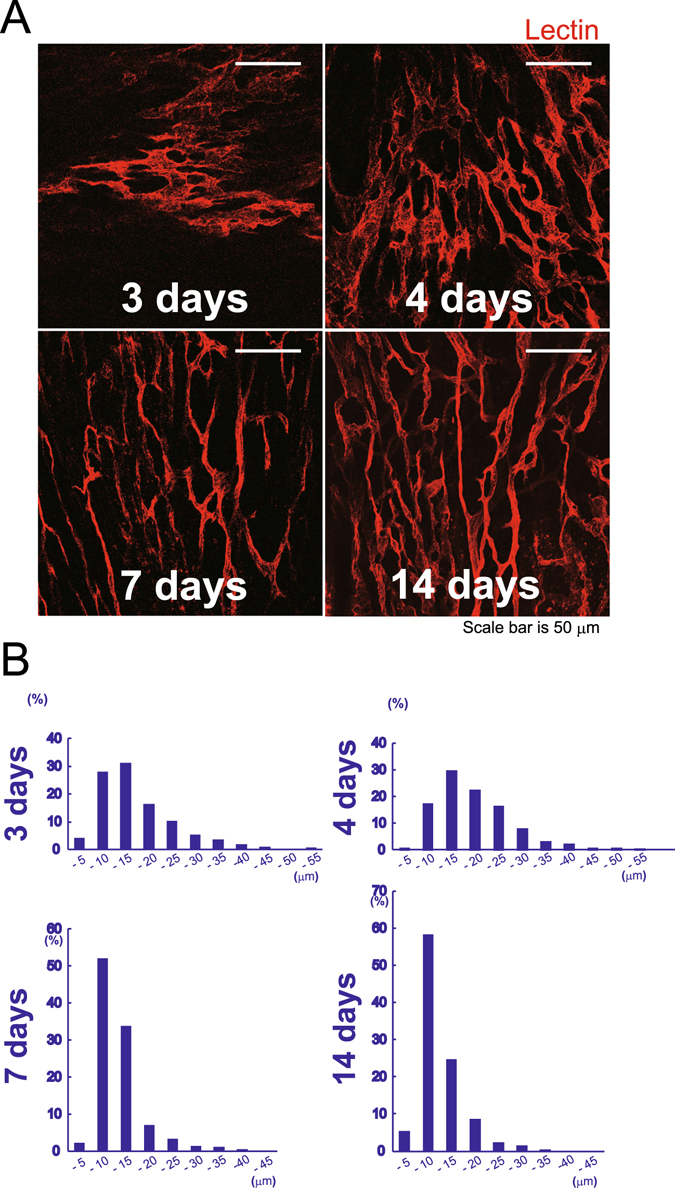
The morphology of the developing vessels was visualized on a plane that was parallel to the endocardium. (**A**) Dilated new vessels formed at abnormal angles between days 3 and 4, and they had different diameters in consecutive segments of a single vessel. The morphology of the vessels altered after day 4, when the vessels became a mature circulatory network with uniform vessel diameters. Vessels were stained with perfused lectin. Scale bar, 50 μm. (**B**) The distribution of the vessel diameters at each time point. The variation was less and the average diameter of the vessels was narrower on day 7 compared with days 3 and 4 (*n* = 7 areas per time point).

To observe the vascular remodeling process, we evaluated the morphology of the newly formed vessels in whole mount tissue samples of the infarct areas. Functional vascular loops in the plane that was parallel to the endocardium were labeled with rhodamine-conjugated lectin and visualized using confocal microscopy. Dilated and short conduits with abnormal branching angles were observed during the early phase of vessel development on day 3 (Fig. 2A). New vessels had rapidly covered an extensive area by day 4, and they consisted of consecutive segments with variable diameters within a single vessel that comprised multiple branches. The morphology of these vessels changed between days 4 and 7, at which time no dilated vessels were observed. A broad range of vessel diameters were observed in the day 3 and day 4 samples compared with those from day 7 and day 14 (Fig. 2B), and >50% of the vessels in the samples from day 7 and day 14 and 23% of the vessels in samples from day 4 were <10 μm in diameter. The median values of diameters at each time point were as follows: day 3: 12.7 μm; day 4: 15.4 μm; day 7: 9.5 μm; and day 14: 8.6 μm (Fig. 2B). This represented the remodeling of a plexus of primitive vessels that later formed the mature circulatory system.

### Newly developed vessels are covered by pericytes

**Figure 3:**
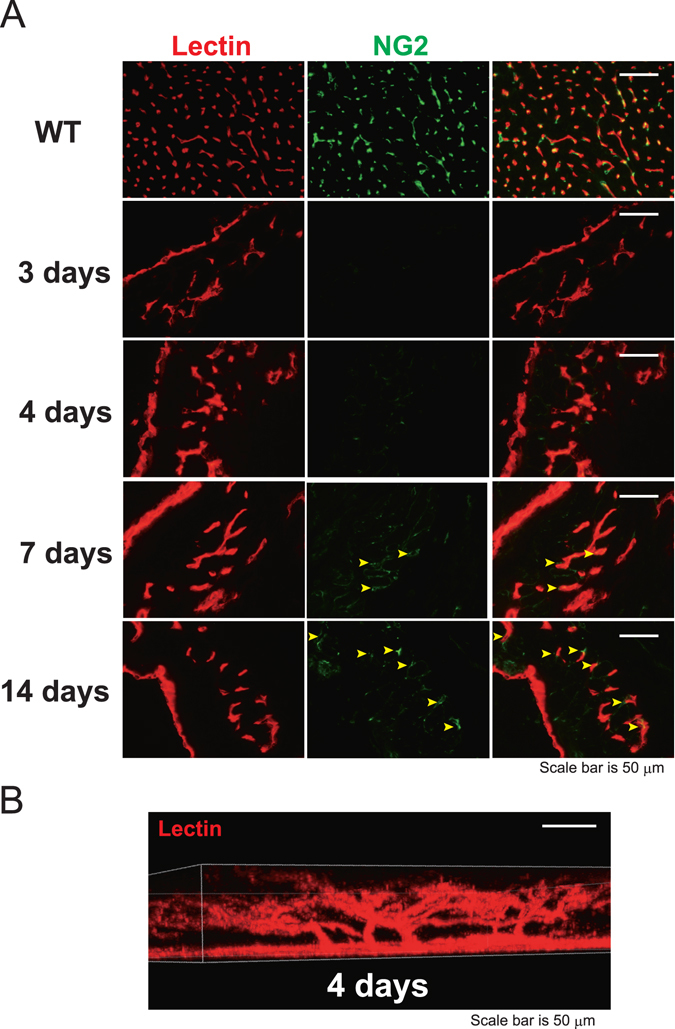
The induction of vessel maturation by the pericytes was evaluated using neural/glial antigen 2 (NG2) staining. (**A**) The new vessels were covered by NG2-positive pericytes after day 7 (arrowheads), but not on days 3 or 4. Scale bar, 50 μm. (*n* = 3 areas per time point) (**B**) The three-dimensional structure of the vessels developing from the left ventricle on day 4. Vessels were stained with perfused lectin. Scale bar, 50 μm.

Nascent vessels are stabilized by recruited mural cells, which provide structural support and regulate vessel function^7, 8^. We used immunohistochemistry to determine whether pericytes were present around the newly formed vessels. On days 3 and 4, no pericytes were present that expressed the integral membrane chondroitin sulfate proteoglycan, neural/glial antigen (NG)2, around the endothelial cells of the new vessels. However, the endothelial cells were covered by pericytes on day 14 (Fig. 3A). These data indicate that primitive vessels mature along with the wall and network structures of blood vessels during the 2 weeks that follow an MI. When we examined the architecture of the new vessels on day 4 using three-dimensional multiphoton microscopy, we observed vascular trees developing from the endocardium (Fig. 3B; Supplementary movie). The architecture of the vessels on day 7 and day 14 were shown in Fig. S2A. Schema of Fig. S2B explains the relations of Figs 1, 2A and 3B.

### Angiogenesis from the endocardium depends on VEGFR2 signaling

**Figure 4:**
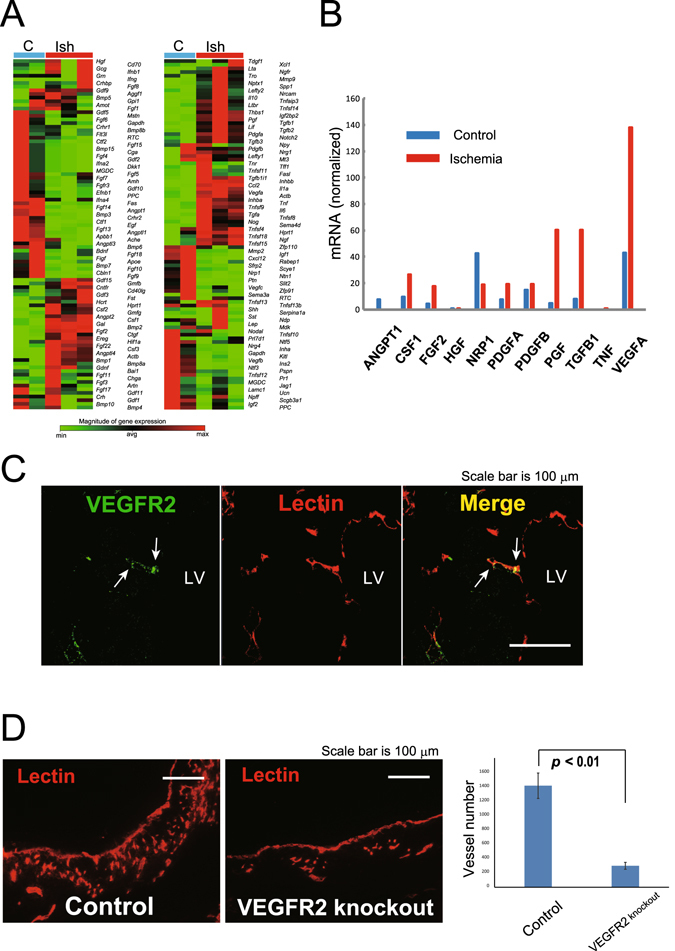
The vascular endothelial growth factor (VEGF)–VEGF-receptor 2 (VEGFR2) axis plays an important role in the development of vessels from the left ventricle (LV). (**A**) Gene expression was examined in a hypoxyprobe-positive area 24 h after myocardial infarction (MI) induction, and the expression of 180 genes was investigated in the hypoxic area. The levels of 39 genes were upregulated by >5-fold in the hypoxic relative to those in the control tissues (ischemic tissues: *n* = 3; control tissues: *n* = 2). (**B**) Expression levels of the representative genes. VEGF-A expression increased markedly in the hypoxic area. (**C**) VEGFR2 was expressed in the primitive vessels of the endocardium on day 3 after MI (arrows). Scale bar, 100 μm. (**D**) The role of VEGFR2 in angiogenesis from the LV. Vessel development was less prominent in the mice that were deficient in VEGFR2 compared with that in the control mice. Vessels were stained with perfused lectin. Scale bar, 100 μm. (control tissues: *n* = 4; knockout tissues: *n* = 4).

To determine which factors modulate vascular development from the endocardium, we examined gene expression in the hypoxyprobe-positive, non-perfused area near the endocardium. Heart tissue samples were collected 24 h after the induction of MI by laser micro-dissection. We used a custom mouse polymerase chain reaction (PCR) microarray to compare the expression of 180 genes in the hypoxyprobe-positive areas of the MI samples with that in the corresponding areas in samples from sham-operated mice that did not have ischemia (Fig. 4A). A total of 39 genes were upregulated by >5-fold and 20 genes were downregulated by <5-fold in the ischemic tissue relative to those in the control tissue. A close examination of the factors that were predicted to play important roles in angiogenesis revealed that the angiopoietin-1 level was markedly reduced, while the levels of VEGF-A and FGF-2 were higher in the ischemic tissue compared with those in the control tissue^9–11^. In particular, VEGF-A showed a high basal level of expression that was strongly induced by ischemia (Fig. 4B). These data indicate that VEGF is a key factor in the development of new vessels from the endocardium.

VEGF-A is a critical regulator of vasculogenesis and angiogenesis during development^12^, and VEGF receptor (VEGFR)2 is essential for endothelial cell function during vascular development and vessel maintenance^13^. We examined the expression of VEGFR2 by newly formed vessels to evaluate the role of VEGF-VEGFR2 signaling in vessel growth. VEGFR2 was expressed by the endothelial cells of preexisting and developing vessels (Fig. S3A), and primitive vessels that had developed from the endocardium clearly expressed VEGFR2 as early as day 3 (Fig. 4C).

To confirm the role of VEGFR2 in angiogenesis from the endocardium, we selectively ablated VEGFR2 expression in the endothelial cells by injecting cadherin5-CreERT2/VEGFR2^loxP/loxP^ mice with tamoxifen over 3 days, which started from day 2 after the induction of the MI. The downregulation of the VEGFR2 levels in the newly formed vessels in the knockout mice was confirmed by immunocytochemistry on day 5 (Fig. S3B). We then compared the vascular development in these mice with that in tamoxifen-treated cadherin5-CreERT2 mice^14, 15^. Five days after MI induction, the new vessels from the endocardium were significantly less prominent in the mice that were deficient in VEGFR2 compared with those in the control animals (Fig. 4D). These data demonstrate that VEGF expression in hypoxic tissue and VEGFR2 expression on primitive vessels are critical for vascular development from the endocardium.

### High oxygen levels increase the survival of cardiomyocytes along the endocardium and suppress cardiac remodeling

**Figure 5:**
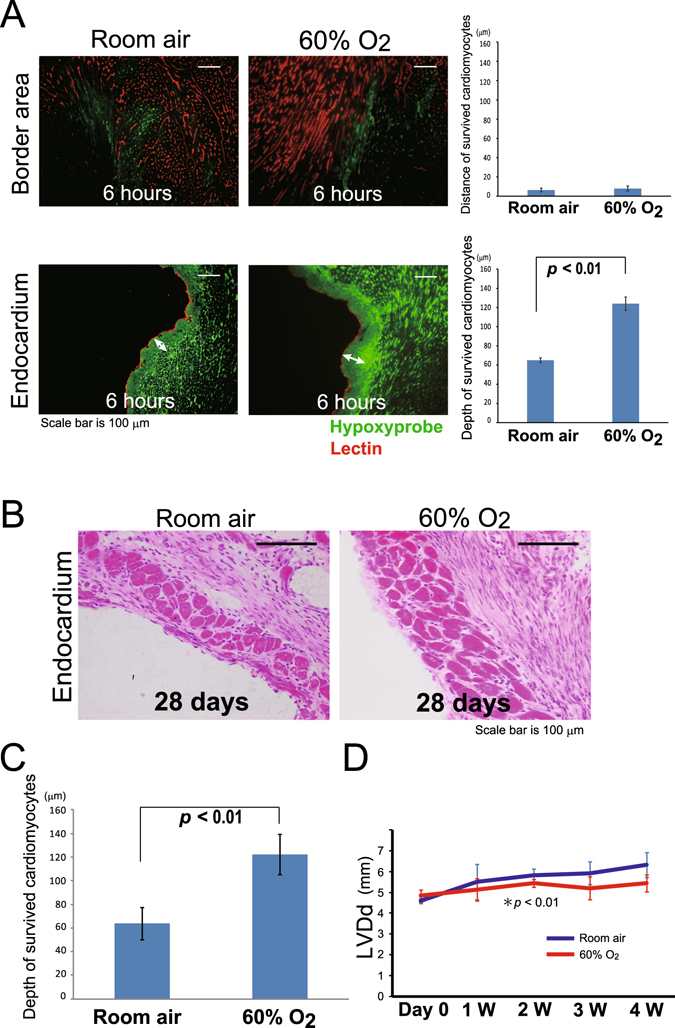
Oxygenation of the infarcted heart increases the number of living cardiomyocytes through vessel development from the left ventricle (LV) and cardiac remodeling is suppressed. The mice were maintained in a chamber of room air or at 60% O_2_ for 4 days after MI induction. (**A**) The hypoxyprobe-positive area increased in the area along the endocardium of the mice that had been exposed to 60% O_2_ at 6 h after MI induction (arrows). Scale bar, 100 μm. (n = 4 per group) (**B**) High concentrations of oxygen (60%) did not affect the hypoxyprobe-positive area in the border area, and resulted in a wider area of surviving cardiomyocytes along the endocardium relative to that in the control group on day 28. Scale bar, 100 μm. (**C**) The distance between the surviving cardiomyocytes and the endocardium was greater in mice that had been exposed to O_2_ compared to those maintained in room air (*n* = 4 per group). (**D**) Remodeling of the infarcted heart was assessed by measuring the left ventricular end-diastolic diameter for 4 weeks. LV dilation was significantly lower in the O_2_-treated group compared with that in the control group (n = 5 per group).

We found that cardiomyocytes along the endocardium can survive because of O_2_ diffusing from the LV for 3 days after MI induction and blood supplied by the new vessels that developed from the LV during the chronic phase of angiogenesis. We hypothesized that high O_2_ levels increase the distance over which O_2_ can diffuse from the endocardium, with the newly formed vessels perfusing a broader area of cardiomyocytes. To examine the effects of O_2_ treatment, mice were maintained in a chamber with 60% O_2_ for 4 days after MI induction, then they were transferred to a room with a normal air environment. At 6 h after MI induction, a larger hypoxyprobe-positive area was observed along the endocardium in the hearts of the mice that had been exposed to 60% O_2_ compared with that in the control mice that had been maintained in room air (Fig. 5A). An environment comprising 60% oxygen did not affect the hypoxyprobe-positive areas in the border area. At 4 weeks after MI induction, there were more surviving cardiomyocytes in the group that had been exposed to 60% O_2_ (Fig. 5B). Cardiomyocytes that were >100 μm away from the endocardium survived in the MI mice that had been exposed to 60% O_2_ for the first 4 days following MI induction, and the thickness of the layer of surviving cardiomyocytes along the endocardium was significantly wider in these mice compared with that in the control mice (Fig. 5C).

Ventricular remodeling after MI is associated with expansion of the infarct zone, time-dependent dilation of the LV, and distortion of the ventricular shape. Infarct expansion refers to the radial thinning and increase in the circumference of a transmural infarct^16^. Ventricular remodeling is the major determinant of the long-term outcomes following MI^17^. As such, treatment approaches that increase the number of cardiomyocytes along the endocardium are promising if the surviving cardiomyocytes in the infarct zone can prevent excessive thinning of the ventricle wall. We evaluated the morphology of the infarcted heart using echocardiography after high-concentration O_2_ therapy, which protects the cardiomyocytes along the endocardium after MI. The left ventricular end-diastolic diameter (LVDd) of the infarcted heart was measured for 4 weeks in mice that had been exposed to 60% O_2_ for the first 4 days after MI induction and in those that had been maintained in room air after MI induction. In the latter group, the LVDd increased in a time-dependent manner, while O_2_ administration significantly suppressed LV dilation (Fig. 5D).

Given that the cardiomyocytes along the endocardium survived because of O_2_ diffusion and the presence of vessels from the LV in this mouse MI model, we examined whether surviving cardiomyocytes were present along the endocardium in a human MI autopsy sample. A layer of live cardiomyocytes were observed around the endocardium in the infarct area (Fig. S4), indicating that the induction of vessel development can also rescue the hypoxic area in the human heart.

### Reversibly damaged cardiomyocytes in the ischemic border area disappear within 12 h

**Figure 6:**
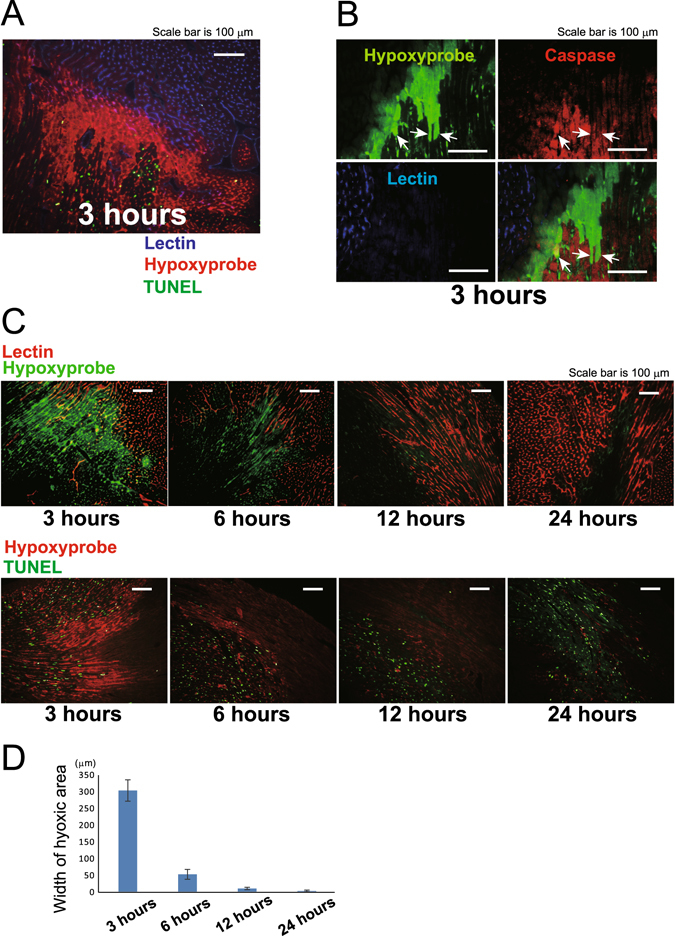
Cardiomyocytes in the border zone are either destined to survive or die within 12 h, and are never in an intermediate state. (**A**) Relationship between lectin, hypoxyprobe, and terminal deoxynucleotidyl transferase-mediated dUTP nick end labeling (TUNEL) staining in 3-h after myocardial infarction induction. The non-perfused TUNEL-negative area was hypoxyprobe-positive. Scale bar, 100 μm. (**B**) A subset of cardiomyocytes distant from the perfused vessels in the hypoxyprobe-positive area expressed caspase at 3 h (arrows). Scale bar, 100 μm. (**C**,**D**) The area within 100–200 μm of the end of the perfused vessels was hypoxyprobe-positive at 3 h; this area diminished in a time-dependent manner and had disappeared by 12 h (n = 4 per time point). Scale bar, 100 μm.

Experimental and clinical studies have targeted cardiomyocytes in the border zone between the perfused and non-perfused areas of the infarcted heart^18^. We investigated the changes in the border zone over time by evaluating the perfused vessels, apoptotic cells, and the hypoxic area after left coronary artery ligation. Lectin was injected intravenously before the animals were sacrificed to detect the perfused vessels only. Apoptotic cells were detected using terminal deoxynucleotidyl transferase-mediated dUTP nick end labeling (TUNEL)^19^, and the hypoxic areas were identified by hypoxyprobe staining, which detects cells with reduced levels of blood perfusion in heart tissue. The cardiomyocytes that were not perfused by blood from the coronary artery and did not undergo apoptosis were hypoxyprobe-positive (Fig. 6A), indicating that the O_2_ tension in tissue without blood perfusion can easily decline to <10 mmHg in the beating heart and that the probe specifically detects cells in this area. The hypoxyprobe does not penetrate cells that are undergoing apoptosis, even in a hypoxic environment; positive cells, therefore, represent reversibly injured cardiomyocytes. We reasoned that the hypoxyprobe-positive area would reduce in size, because cardiomyocytes in the hypoxic area undergo apoptosis in a time-dependent manner. We assessed intracellular caspase activation using pan-caspase immunolabeling for caspases 1, 2, 3, 6, 7, 8, 9, 10, and 13 to determine whether caspases are activated in hypoxic cardiomyocytes before deoxyribonucleic acid (DNA) strand breakage occurs. A subset of cardiomyocytes at a distance from the perfused vessels in the hypoxyprobe-positive area expressed caspase (Fig. 6B), and these were considered to be in the early stages of apoptosis. These results indicate that the hypoxyprobe-positive area contained live, reversibly damaged cardiomyocytes that could potentially be rescued. The size of this border zone declined in a time-dependent manner between 3 h and 12 h after MI induction (Fig. 6C,D). The areas within 100–200 μm from the ends of the perfused vessels were hypoxyprobe-positive in the 3-h samples, whereas no signal was observed in the border zones of the 12-h and 24-h samples. This suggests that the cardiomyocytes in this zone are destined to either survive when blood perfusion is present or die within 12 h when it is absent, and that they are never in an intermediate state 12 h after an MI. Therefore, therapies based on anti-apoptotic or pro-angiogenic factors would not affect the border zone between the perfused and the non-perfused areas 12 h after an MI, because this area has stabilized within this time frame.

## Discussion

A variety of new therapies have been pursued to mitigate cardiac damage after ischemic events. Most newly developed therapies that involve angiogenic, anti-apoptotic factors, or specific cells have targeted the interface between the perfused and the non-perfused areas in the border zone, and they have tried to inhibit cardiomyocyte apoptosis or induce angiogenesis in this area. However, no convincing evidence exists that suggests these new therapies are therapeutically beneficial in the ischemic border zone. We need to find the target area that is amenable to treatment and determine the timeframe in which treatment can be beneficial; hence, the physiological processes and the relationships among perfusion, ischemia, and apoptosis in the context of MI must be evaluated in detail.

First, we examined the ischemic area along the endocardium where a few layers of cardiomyocytes that had survived were present, even during the chronic phase of the MI. The area within 100–150 μm of the endocardium was hypoxic with a partial pressure of oxygen that was <10 mmHg for 2 days after the MI, which was confirmed by the hypoxyprobe staining. Surprisingly, primitive vessels developed from the endocardium on day 3 within the ischemic area and they perfused the hypoxic area by circulating oxygenated blood within the LV, which was a new circulatory system that was independent of the coronary circulation. Three-dimensional imaging showed the growth of new vessels from the endocardium that perfused blood in the LV into the heart tissue. Interestingly, primitive vessels with different diameters changed their morphology during vessel remodeling, and they were covered by pericytes during the maturation phase of vessel development. A publication from an embryological study reported that ventricular endocardial cells are angiogenic and form coronary endothelial networks through VEGF-VEGFR2 signaling during mouse development^20^, and it described a source of the coronary endothelial cells and a potential differentiation pathway for the endocardium. These data support our finding that the new vessels that perfused the heart tissue developed from the endocardium under pathological conditions.

We then evaluated the ischemic border zone, which is the interface between the perfused and the non-perfused areas in an MI. The areas within 100–200 μm of the ends of the perfused vessels in the heart were hypoxyprobe-positive 3 h after the MI. The cardiomyocytes that were present in this hypoxic area were not perfused, but they were alive. We suggest that this area is designated as the real border zone in MI. The hypoxyprobe-positive area diminished in a time-dependent manner and it had disappeared by 12 h after the MI. These data show that the cardiomyocytes in the border zone were destined to die within 12 h. Hence, any treatments, including reperfusion, anti-apoptotic, or pro-angiogenic therapies, will be ineffective in this border zone area 12 h after an MI.

Most therapies have attempted to salvage the cardiomyocytes in the border zone area. Our data show that the ischemic border zone area must be the most attractive therapeutic target if therapy begins within 12 h of an MI. On the other hand, the hypoxic area along the endocardium that has no blood perfusion offers great potential for innovative therapies, even during the sub-acute and chronic phases of an MI.

VEGF is one of the most powerful and important angiogenic growth factors. Therefore, we examined the role of VEGF-VEGFR2 signaling in the development of new vessels from the endocardium. The expression of 39 of 180 genes was upregulated by over five-fold in the hypoxyprobe-postive area compared with that in the control tissue. In particular, the expression of VEGF-A increased markedly in the hypoxic area 24 h after an MI was induced. Next, we evaluated the role of VEGFR2 in angiogenesis from the LV, and we found that VEGFR2 was expressed at an early stage of angiogenesis, even in the primitive vessels of the endocardium. To confirm the importance of VEGF-VEGFR2 signaling, we suppressed the expression of VEGFR2 on the endothelial cells of the primitive vessels using tamoxifen-induced conditional knockout mice, and found that the new vessels originating from the endocardium were less prominent in the mice that were deficient in VEGFR2 compared with those in the control mice 5 days after the MI. The new circulatory system that is derived from the ventricle is highly dependant on VEGF-VEGFR2 signaling. In our experiment new vessels from the endocardium were significantly suppressed in the mice that were deficient in VEGFR2. We speculate that both steps of sprouting and development were suppressed in VEGFR2 deficient mice.

Finally, we investigated the possibility of applying therapy to the ischemic area along the endocardium in the infarcted heart. We observed that a thin zone along the endocardium in the area with no perfusion survived when oxygen diffused from the LV, even though the zone was hypoxic. To expand the area that was oxygenated from the LV, we applied a higher concentration of oxygen from 1 h to 4 days after the MI was induced; the latter timepoint being when the new vessels from the endocardium had grown sufficiently to oxygenize this area. Compared with the mice that were maintained in room air, treatment with 60% oxygen enhanced oxygen diffusion from the ventricle and augmented the hypoxyprobe-positive area along the endocardium during the early phase of MI, and a wider area of surviving cardiomyocytes was present on day 28. The dilation of the LV, which comprises part of the remodeling of a heart that has undergone MI, was suppressed in the oxygen-treated mice. This finding indicates that the area along the endocardium that contained the surviving cardiomyocytes is an important target for the treatment of MI from the acute phase to the chronic phase. Therapies that suppress apoptosis or induce angiogenesis in this particular area could be applied in combination with therapy comprising a high concentration of oxygen as was applied in this study.

Moreover, regenerative medicine has been evolving dramatically since the discovery of inducible pluripotent stem cells^21^. Identifying the conditions in which stem cells can survive and differentiate into mature cells is crucial for the beneficial application of regenerative cells to the infarcted heart^22^. We consider that undifferentiated cells could engraft and proliferate in the area along the endocardium, and that deeper knowledge about this area could contribute to the evolution of regenerative medicine in ischemic heart disease.

The notion that the cardiomyocytes along the endocardium can survive in the infarcted area was confirmed in an autopsy sample from a patient who had experienced an MI. Hence, we should pursue the potential clinical relevance of this particular area to ischemic heart disease.

Here we have verified the presence of a novel circulatory system that develops from the LV, which is separate from the coronary circulation, during the pathological conditions associated with MI. Furthermore, our results indicate that the area along the endocardium that is perfused by blood from the LV could be the target for the future treatment of the acute and chronic phases of MI.

## Materials and Methods

### Mice

Eight-week-old male C57BL/6 J mice were purchased from SLC Co. Ltd. (Nagoya, Japan). Cadherin5-CreERT2 mice were crossed with mice carrying a loxP-flanked *Vegfr2* gene (*Vegfr2* ^*loxP*/loxP^)^14, 15^. To induce Cre activity in the adult mice, three intraperitoneal injections of tamoxifen (50 μl) were administered on days 2, 3, and 4 after MI induction. The phenotype of the conditional mutant mice with MIs was analyzed on day 5.

All experimental procedures and animal-care protocols were approved by the Institutional Animal Care and Use Committee of Nagoya University. All experiments were carried out in accordance with the approved protocols and guidelines.

### MI model

MI was induced as previously described^23^. Briefly, the animals were anesthetized with an intraperitoneal injection of pentobarbital (50 mg/kg), orally intubated with a 22 intravenous catheter, and provided with ventilation through a respirator (Harvard Apparatus, Holliston, MA, USA). A small skin incision was made, a left intercostal thoracotomy was performed at the fourth intercostal space, and the ribs were separated using 6–0 polypropylene sutures to expose the heart. The pericardium was opened and the left anterior-descending (LAD) branch of the left coronary artery was ligated proximal to the bifurcation between the LAD and the diagonal branch using an 8–0 polypropylene suture. Positive end-expiratory pressure was applied to fully inflate the lung, and the ribs, minor and greater pectoral muscles, and skin were closed with a 6–0 polypropylene suture.

### Assessment of cardiac function

Cardiac function was evaluated with a commercially available high-resolution echocardiographic system equipped with a 30-MHz mechanical transducer (VEVO700; Fujifilm VisualSonics, Amsterdam, Netherlands). The mouse was secured to a warming platform in the supine position with electrodes taped to the limbs, and the thorax was shaved and cleaned with a chemical hair remover to minimize ultrasound attenuation. Aquasonic 100 gel (Parker Laboratories, Inc., Fairfield, NJ, USA), from which all of the air bubbles had been extruded, was applied to the thorax to optimize the visibility of the cardiac chambers. The images were evaluated by planimetry, as recommended by the manufacturer. Two-dimensional, parasternal long- and short-axis views were acquired, and the endocardial area was calculated by tracing the endocardial limits of each frame in the long-axis view, with the minimal and maximal areas designated as the LV end-systolic volume (LVESV) and the end-diastolic volume (LVEDV), respectively. The system’s software uses a formula based on a cylindrical-hemiellipsoid model (volume = 8.area²/3π/length) to calculate the LV ejection fraction (LVEF) and the stroke volume (SV) as follows: LVEF = (LVEDV − LVESV)/LVEDV × 100. The SV was defined as (aortic velocity time integral) × π(aortic valve diameter/2)^2, 24^. The LVEF and SV were calculated for three cardiac cycles per mouse per time point, and the average of the three cycles is reported.

### Histological analysis

To stain sections for lectin, the mice were intravenously perfused with 250 μg *Griffonia simplicifolia* lectin I (Vector Laboratories, Burlingame, CA, USA) for 30 min before they were euthanized to identify functional microvessels^25^. The heart was dissected and snap frozen in liquid nitrogen, and cut into 7-μm sections that were fixed with 4% paraformaldehyde. The sections were labeled with a goat anti-lectin antibody (Vector Laboratories), which was followed by labeling with Alexa Fluor^®^-conjugated secondary antibodies (Invitrogen, Carlsbad, CA, USA). For whole-heart lectin staining, the mice were perfused with 250 μg of rhodamine-conjugated *G. simplicifolia* lectin I (Vector Laboratories)^26^. The heart was dissected and the infarct area was mounted on a microscope slide using Vectashield^®^ (Vector Laboratories) after removing the non-infarcted areas.

The Hypoxyprobe-1 Omni kit (Hypoxyprobe, Inc., Burlington, MA, USA) was used to detect the hypoxic areas according to the manufacturer’s protocol. Briefly, 1 h before euthanasia, the mice were intraperitoneally injected with a 10 mg/mL solution (60 mg/kg) of hypoxyprobe-1^27, 28^, which is a water-soluble substituted 2-nitrominidazole that is widely used to detect hypoxic conditions in live tissues and cell cultures. Once injected into an animal, the probe is rapidly distributed to all of the tissues in the body, but it only forms adducts with proteins in cells that have O_2_ concentrations <14 μM, which is equivalent to a partial pressure of O_2_ of 10 Torr at 37 °C. The mice were sacrificed by cervical dislocation to abruptly cut off the blood circulation. The heart was dissected and snap frozen in liquid nitrogen, and cut into 7-μm sections that were fixed with 4% paraformaldehyde. The sections were labeled with the rabbit anti-hypoxyprobe antibody that is included in the kit, followed by labeling with an Alexa-Fluor^®^-conjugated secondary antibody.

To assess the maturity of the new vessels, the vascular pericytes were identified using NG2 immunocytochemistry^29^. The tissue sections were fixed with 4% paraformaldehyde and then they were labeled with a polyclonal anti-NG2 antibody (Merck Millipore, Billerica, MA, USA), which was followed by labeling with an Alexa-Fluor^®^-conjugated secondary antibody.

### Detection of apoptosis

Cell death was detected *in situ* using a TUNEL staining^30^ kit (Roche Diagnostics, Indianapolis, IN, USA) according to the manufacturer’s instructions. The sections were fixed with 4% paraformaldehyde and treated with a permeabilization solution (0.5% Triton X-100) for 2 min on ice. After washing, the labeling reaction was carried out using a solution containing terminal deoxynucleotidyl transferase, buffer, and fluorescein-dUTP.

Poly-caspase FLICA SR-VAD-FMK reagent (ImmunoChemistry Technologies, LLC, Bloomington, MN, USA), which detects most active caspases (caspase-1, -3, -4, -6, -7, -8, and -9), was used according to the manufacturer’s instructions^31^. The sections were fixed in cold acetone and they were incubated in the FLICA reagent working solution for 1 h, which was followed by washing with 0.05% Tween 20. The cell-permeant poly-caspase FLICA SR-VAD-FMK reagent forms irreversible bonds with activated intracellular caspases, and the complex is retained by cells during washing.

### Fluorescence imaging

To assess vessel perfusion, the hypoxic areas, apoptosis, and pericyte coverage, cryosections were processed and were visualized and imaged using an all-in-one microscope system (BZ8000; Keyence Corporation, Osaka, Japan) with a 20× objective lens (Plan Fluor 20×: numerical aperture [NA] = 0.75; working distance = 1.0 mm; Nikon Corporation, Tokyo, Japan).

The infarct area that was stained with rhodamine-conjugated *G. simplicifolia* lectin I was placed on a microscope slide with Vectashield^®^ and it was flattened with a cover slip. New vessels in the infarct area were imaged using confocal microscopy (LSM 5 Pascal; Carl Zeiss AG, Oberkochen, Germany) by scanning 12 layers (with 2.5 μm between the adjacent layers) using a 20 × objective lens (EC Plan-Neofluar 20 × : NA = 0.5; working distance = 2.0 mm; Carl Zeiss AG).

### Three-dimensional two-photon microscopy

The infarct area that was stained with rhodamine-conjugated *G. simplicifolia* lectin I was placed on a microscope slide with Vectashield^®^ and flattened with a cover slip. The infarct area was imaged using an upright multiphoton microscope (A1RMP; Nikon Corporation) to visualize the complete structure of the vessels using a 25 × objective lens (CFI Apo LWD 25 × W: NA = 1.1; working distance = 2.0 mm; refractive index = 1.33; Nikon Corporation). MaiTai HP DeepSee^®^ (Spectra-Physics, Santa Clara, CA, USA) was used for the two-photon excitation of rhodamine. Nikon NIS-Elements software was used to generate three-dimensional reconstructions from z-stacks for each fluorescent signal, which were 512 × 512 pixels in the x-y image plane and typically comprised 60–80 slices (1 slice per μm^−1^) in the z dimension.

### Vessel measurements

Vessel diameter was measured in a whole-mounted infarct area that was stained with rhodamine-conjugated *G. simplicifolia* lectin I by overlaying representative phase images with a computer-generated square lattice. Squares were selected at random and the diameter of each vessel in the center of each of the selected squares was measured^32^.

### Laser microdissection, ribonucleic acid isolation, and gene expression analysis

Heart tissue samples from three MI mice that had been sacrificed 24 h after coronary ligation and from two sham-operated mice were prepared as described next. Serial 10 μm-cryostat sections were mounted on polyethylene naphthalate membrane slides (Leica Camera AG, Wetzlar, Germany). The hypoxic areas along the endocardium of the MI samples, which had been identified in the images from the serial sections stained with the hypoxyprobe, were visualized with a Leica LMD7000 system (Leica Camera AG) without fixation or staining^33^. Non-ischemic areas along the endocardium of the sham-operated mice were also dissected. Tissue samples were transferred by gravity into the cap of a microcentrifuge tube placed underneath the section. The tube cap contained 50 μl extraction buffer from the PicoPure RNA Isolation kit (Life Technologies, Carlsbad, CA, USA)^34^. About 3 mm^2^ of the cut tissue was collected for each sample. After incubation at 42 °C for 30 min, the total ribonucleic acid (RNA) was extracted from the tissue lysate using the PicoPure RNA isolation kit according to the manufacturer’s protocol. Briefly, the RNA purification column was prepared with conditioning buffer, and 50 μl of 70% ethanol were added to the sample solution and the mixture was applied to the preconditioned column. This was followed by DNase treatment, two washes, and RNA elution with 11 mL of the elution buffer. The RNA solution was treated with the RT2 Nano Pre Amp cDNA Synthesis kit (SA Biosciences, Frederick, MD, USA) according to the manufacturer’s instructions to obtain complementary DNA (cDNA)^35^. Pre-amplified cDNA was mixed with RT^2^ SYBR Green/ROX qPCR master mix (SA Biosciences) and 25 μl aliquots were loaded into each well of a custom-designed PCR array. A real-time quantitative reverse transcription-PCR was carried out using a Stratagene Mx3000 quantitative PCR system (Agilent Technologies, Santa Clara, CA, USA). The amplification conditions were as follows: 10 min at 95 °C, followed by 40 cycles of 15 s at 95 °C and 1 min at 60 °C. A dissociation curve from 65 °C to 95 °C was generated for each plate immediately after the PCR to determine the quality of the specific products amplified in each well. The resultant threshold cycle values for each well were analyzed using the RT^2^ Profiler PCR Array Data Analysis Template file provided by SA Biosciences.

### Exposure to oxygen

The mice were transferred to a breeding chamber (Deuce Corporation, Pekin, IL, USA) that was coupled to an O_2_ cylinder through an O_2_ controller ProOx110 (Biospherix Ltd., Lacona, NY, USA) within 1 h of MI induction^36^. The mice were maintained in the chamber in which the O_2_ concentration was 60%, for 4 days with free access to water and food.

### Histological analysis of human myocardial infarction heart tissue specimens

Hearts that had undergone MI were obtained from autopsies, and were fixed in 10% neutral-buffered formalin and embedded in paraffin. The scarred areas were examined by staining 5-μm-thick sections with hematoxylin and eosin.

### Statistical analysis

Data are presented as mean ± SEM. Comparisons between two groups were evaluated for significance with the Student t-test and measurements obtained in the same animal at multiple time points were evaluated via two-way ANOVA analysis. A *P* value less than 0.05 was considered significant.

## Electronic supplementary material

Supplementary Figures

Supplementary movie
