# Supplementary material for: Dynamics of angiogenesis in ischemic areas of the infarcted heart
Source: Sci Rep. 2017 Aug 2;7:7156. doi: 10.1038/s41598-017-07524-x (PMC5540926; doi:10.1038/s41598-017-07524-x)
Supplement: Supplementary file 1 — Supplementary Figures [file 41598_2017_7524_MOESM1_ESM.doc]

**Dynamics of angiogenesis in ischemic areas of the infarcted heart**

Koichi Kobayashi^1^*, Kengo Maeda^1^, Mikito Takefuji^1^, Ryosuke Kikuchi^2^, Yoshihiro Morishita^1^, Masanori Hirashima^3^, & Toyoaki Murohara^1^

**Supplementary Figure 1. The relation of hypoxic area and apoptosis in the area along endocardium (A)** The vessels which are not perfused by the coronary ligation were not stained with lectin, but are stained with CD31 endothelial marker 6 hours after MI induction.

Scale bar, 100 μm. **(B)** Hypoxyprobe negative area in non-perfusion area showed caspase activitivation. Scale bar, 100 μm. **(C)** Relationship between hypoxyprobe and terminal deoxynucleotidyl transferase-mediated dUTP nick end labeling (TUNEL) staining. Hypoxyprobe positive area along endocardium did not undergo apoptosis. Scale bar, 100 μm..

**Supplementary Figure 2. The architecture of newly formed vesssels from endocardium**

**(A)** 3D structure of new vessels developing from endocardium on day 7 and 14. Scale bar, 50 μm. **(B)** Vascular trees were observed in the plane that was parallel or perpendicular to the endocardium.

**Supplementary Figure 3. Vascular endothelial growth factor-receptor 2 (VEGFR2) expression in primitive vessels and in conditional knockout mice. (A)** VEGFR2 was expressed by the endothelial cells of preexisting and primitive vessels that had developed from the endocardium. Scale bar, 100 μm. **(B)** The expression of VEGFR2 was downregulated on newly formed vessels in knockout mice. Scale bar, 100 μm.

**Supplementary Figure 4. Human samples from patients who experienced myocardial infarctions.** Living cardiomyocytes were present along the endocardium in the ischemic areas of human infarcted heart tissue samples. Scale bar, 100 μm.
